# Molecular Mechanisms in the Transformation from Indolent to Aggressive B Cell Malignancies

**DOI:** 10.3390/cancers17050907

**Published:** 2025-03-06

**Authors:** Nawar Maher, Samir Mouhssine, Bassam Francis Matti, Alaa Fadhil Alwan, Gianluca Gaidano

**Affiliations:** 1 Division of Hematology, Department of Translational Medicine, Università del Piemonte Orientale and Azienda Ospedaliero-Universitaria Maggiore della Carità, 28100 Novara, Italy; nawar.maher@uniupo.it (N.M.); samir.mouhssine@uniupo.it (S.M.); 2 Department of Hematology and Bone Marrow Transplant, Hematology and Bone Marrow Transplant Center, Medical City, Baghdad 00964, Iraq; bassam_francis@yahoo.com; 3 Department of Clinical Hematology, The National Center of Hematology, Mustansiriyah University, Baghdad 10001, Iraq; ala_sh73@uomustansiriyah.edu.iq

**Keywords:** chronic lymphocytic leukemia, follicular lymphoma, lymphoplasmacytic lymphoma, marginal zone lymphoma, Richter transformation, diffuse large B cell lymphoma, histological transformation

## Abstract

Histological transformation (HT) into aggressive lymphoma is a turning point in a significant fraction of patients affected by indolent lymphoproliferative neoplasms, namely, chronic lymphocytic leukemia (CLL), follicular lymphoma (FL), marginal zone lymphomas (MZLs), and lymphoplasmacytic lymphoma (LPL). The most common histologic subtype resulting from HT is diffuse large B cell lymphoma (DLBCL). Consistently, DLBCL arisen from indolent non-Hodgkin lymphomas still represents an unmet clinical need, due to disease aggressiveness and resistance to available treatment approaches. Numerous molecular alterations associated with the occurrence of the aggressive HT of CLL (known as Richter transformation) have been identified, namely, genetic lesions of *TP53*, *CDKN2A*, *NOTCH1*, and *c-MYC*, dysregulation of immune checkpoints, and hyperactivation of the B cell receptor pathway. HT of FL involves a combination of genetic and epigenetic alterations affecting genes involved in cell-cycle control (e.g., *CDKN2A/B*), DNA damage response (e.g., *TP53* and *BCL6*), proliferation including the NF-κB pathway, and microenvironmental interactions (namely, *TNFSRF14*). The molecular pathways driving MZL transformation include copy number alterations, mutations of *NOTCH2*, *KLF2*, *TNFAIP3*, *KMT2D*, and *FOXP1*, as well as epigenetic dysregulation. The molecular alterations underlying the aggressive transformation of LPL are poorly understood, with *CXCR4* mutations being the primary factor involved. Current knowledge of the molecular mechanisms of HT has led to promising therapeutic strategies targeting immune checkpoints, BCL2, and BTK, and exploiting bispecific monoclonal antibodies and chimeric antigen receptor (CAR)-T cells. Further investigations are necessary for a deeper understanding of the molecular mechanisms underlying HT, in order to further highlight predictive biomarkers of transformation and develop effective treatment strategies. The aim of this review is to provide a comprehensive overview of the molecular mechanisms that drive transformation to DLBCL of CLL, FL, MZLs, and LPL.

## Simple Summary

A significant fraction of patients affected by indolent B cell malignancies, such as chronic lymphocytic leukemia, follicular lymphoma, marginal zone lymphoma and lymphoplasmacytic lymphoma, may experience a transformation into a more aggressive form of lymphoma, often referred to as diffuse large B cell lymphoma. This transformation poses significant treatment challenges due to the increased aggressiveness of the disease and its resistance to available therapies. This review aims to explore the molecular mechanisms underlying this transformation, identifying genetic alterations and immune system changes that contribute to the progression of the disease. These changes affect several processes such as cell growth, DNA repair, and immune response. Understanding these mechanisms is crucial for developing targeted treatments and improving patient outcomes. This work provides an overview of the complex processes behind the transformation of these tumors, highlighting the need for continued investigation to enhance treatment strategies and patient care.

## 1. Introduction

Histological transformation (HT) into aggressive lymphoma is a turning point in a significant fraction of patients affected by indolent lymphoproliferative neoplasms, namely, chronic lymphocytic leukemia (CLL), follicular lymphoma (FL), marginal zone lymphomas (MZLs), and lymphoplasmacytic lymphoma (LPL) [1,2,3,4]. The most common histologic subtype resulting from HT is diffuse large B cell lymphoma (DLBCL), characterized by even worse clinical outcomes compared to its *de novo* counterpart [5]. DLBCL arisen from indolent non-Hodgkin lymphomas (NHLs) still represent an unmet clinical need, due to the high degree of resistance to available treatment approaches [5]. Although HT of lymphomas was first described more than 80 years ago by Gall and Mallory [6], the biological events that characterize transformation remain incompletely understood, and many questions are still unanswered. Here, we aim to provide a comprehensive overview of the molecular mechanisms that drive DLBCL transformation of CLL, FL, MZLs, and LPL.

### 1.1. Chronic Lymphocytic Leukemia

According to the fifth edition of the *WHO Classification of Haematolymphoid Tumours*, CLL is a B cell neoplasm characterized by the proliferation and accumulation of mature B lymphocytes in blood, lymphoid organs, and eventually, in peripheral tissues [7,8]. CLL clonal lymphocytes express CD19, CD20, CD5, CD23, surface immunoglobulin, CD22, CD79b, CD43, and CD200 [7,8]. CLL is the most frequent leukemia in the adult population, accounting for 4.6 new cases per 100,000 people per year in the US [9]. The slow progression of the disease, combined with advancements in targeted therapies in recent years, has led to relatively long survival rates, with 88.5% of cases achieving a 5-year relative survival [9,10]. A rare variant of the disease with predominant lymph node involvement is known as small lymphocytic lymphoma, which displays similar biological and molecular features as CLL. In the past, chemo-immunotherapy (CIT) was the only treatment option for CLL [11,12]. However, the introduction of pathway inhibitors, namely, Bruton tyrosine kinase (BTK) and B cell lymphoma 2 (BCL2) inhibitors, now enables prolonged progression-free survival (PFS), even in patients with high-risk characteristics such as *TP53* abnormalities and unmutated immunoglobulin heavy-chain variable (IGHV) gene status [13,14].

The most serious complication during the clinical course of CLL is Richter transformation (RT), which is characterized by the development of an aggressive type of lymphoma in CLL patients [1,7,8]. In the CIT era, RT occurred in 2–10% of CLL cases, with a higher development rate in patients who had received previous treatment [1,15]. Conversely, recent data by Hampel et al. suggest that the incidence of RT is significantly lower in patients treated with pathway inhibitors compared to those treated with CIT [16]. The reduced incidence of RT with pathway inhibitors may stem from their ability to minimize genotoxic stress, suppress aggressive subclones, modulate the microenvironment, and/or directly target the molecular pathways driving CLL progression and transformation [16,17]. RT is classified into two different histologic types: DLBCL-type and Hodgkin lymphoma (HL)-type [7,8]. DLBCL-type RT, characterized by confluent sheets of large neoplastic post-germinal center CD20+ B lymphocytes, accounts for 90% of RT cases and is more severe due to its high level of chemo-refractoriness [18,19]. Several studies reported that 70–80% of DLBCL-type RT are classified as non-germinal center B cell (non-GCB) subtype, as determined by the Hans algorithm [20,21]. However, unlike in *de novo* DLBCL, the cell of origin (COO) does not appear to affect clinical outcomes in DLBCL-type RT [22]. Approximately 80% of DLBCL-type RT result from the direct transformation of existing CLL clones, while 20% show different IGHV rearrangements, documenting a *de novo* DLBCL development [1,15]. Clonally unrelated cases tend to have a more favorable prognosis, emerging as secondary malignancies due to immune suppression in CLL, as demonstrated by ultra-deep next generation sequencing (NGS) [23]. Indeed, effective treatment for clonally related DLBCL-type RT remains an unmet medical need, as survival is extremely limited, with a median between 6 and 12 months [24]. Therefore, understanding the molecular mechanisms driving this aggressive transformation is crucial for future directions of tailoring treatment approach.

### 1.2. Follicular Lymphoma

FL is the second most common type of non-Hodgkin lymphoma (NHL) in the USA and Europe (20–25% of NHLs), with approximately 15,000 newly diagnosed cases per year [2,8]. It is considered the prototype of indolent lymphomas and has an equal distribution between males and females and a median age of 65 years at presentation [2]. The translocation t(14;18) (q32;q21), the genetic hallmark of FL, places the antiapoptotic *BCL2* oncogene under the control of the IGH enhancer and is present in the overwhelming majority of cases [25]. FL is a GCB lymphoma characterized by the expression of monoclonal immunoglobulins, CD19, CD20, CD10, and BCL-6 and is negative for CD5 and CD23 [26]. Given the indolent course of FL, 50% of patients are diagnosed with advanced stage and bone marrow involvement. In spite of the improvement in the diagnostic accuracy and the efficacy of CIT regimens, progression and HT still occur [27]. In fact, the rate of transformation to a more aggressive form of lymphoma is 2–3% per year. The most common histologic subtype during transformation is DLBCL, but other histological subtypes may also be seen [28]. The clonal evolution of DLBCL-type transformed FL (tFL) generally follows two primary models: (*i*) the linear model, where the transformed clone arises directly from the indolent lymphoma by acquiring additional genetic alterations, while retaining the genetic abnormalities of the indolent phase [29,30,31]; (*ii*) the divergent/branching model, in which both the indolent FL and the related transformed clones stem from a common progenitor clone that acquires distinct genetic changes independently at each stage [30,31]. Whole-exome sequencing analysis has revealed that the majority of tFL cases (~90%) exhibit a divergent evolution [31]. The introduction of rituximab, an anti-CD20 monoclonal antibody (mAb), has improved the 5-year OS post-transformation to 73% using CIT regimens [32]. Understanding the molecular pathways underlying transformation process can pave the way for the discovery of new therapeutic agents to further enhance treatment outcomes.

### 1.3. Marginal Zone Lymphomas

MZLs arise from mature B cells originating from the marginal zone of lymph nodes, which surrounds the mantle zone of the germinal center [8,33]. MZLs make up 5% to 10% of NHLs. MZLs are classified into three groups: splenic MZL (SMZL), nodal MZL (NMZL), both rare (less than 2% of all NHL), and MZL of mucosa-associated lymphoid tissue (MALT), which represents the most commonly occurring subtype of MZLs [34]. All subtypes of MZLs share morphologic and immunophenotypical similarities, although molecular and clinical characteristics differ for each entity [35]. MZLs generally have an indolent clinical course and a long natural history, with median survival approximating a decade. HT to an aggressive lymphoma is a rare event that can occur in any subtype [36]. The median time to HT in several series ranges from 1 to 15 years after diagnosis, with a frequency of about 2.4% per year, 5% at 5 years, and 10% at 12 years. One of the largest analyses on the frequency and clinical characteristics of transformed MZLs by Conconi et al. reported HT occurrence in 5% of SMZL, 4% of MALT lymphomas, and 3% of NMZL, which is similar to other series, suggesting that the risk of developing HT is similar across the different histological subtypes of MZL [3]. Limited data are available regarding the dysregulated biological pathways involved in the transformation of MZLs, with most studies primarily focusing on SMZL [33]. Transformed MZL is most commonly associated with DLBCL of the non-GCB type, often characterized by dual expression of c-MYC and BCL2 (double expressor phenotype) [3].

### 1.4. Lymphoplasmacytic Lymphoma

LPL is an indolent NHL marked by the clonal proliferation of small, mature B lymphocytes, plasma cells, and lymphocytes with plasmacytoid differentiation, primarily in the bone marrow but potentially affecting other lymphoid tissues and, rarely, extra-lymphoid tissues [7,8]. Two different subtypes of LPL are recognized: (*i*) Waldenström macroglobulinemia (WM) type, which accounts for 95% of the cases and is characterized by the association with IgM paraproteinemia; and (*ii*) non-WM type, a rare subtype representing 5% of LPL, which can be divided into cases with IgG or IgA paraproteinemia, non-secretory LPL, and IgM LPL without bone marrow involvement [8,37]. More than 90% of LPL cases harbor the hallmark activating mutation L625P of the *MYD88* gene, which sustains the survival of malignant B cells [29,38]. The LPL estimated incidence in the US is 0.63 new cases per 100,000 persons per year, but it varies by ethnicity, with northern Sweden having the highest reported incidence globally at 1.75 new cases per 100,000 persons per year [39]. The treatment of LPL has evolved over the years, with a reduced emphasis on CIT, and a greater focus on targeted therapies, particularly BTKi [10]. LPL patients can be further classified into four subgroups based on *MYD88* and *CXCR4* mutations, which predict their response to covalent BTKi: (*i*) *MYD88*mut*CXCR4*wt, the most common subtype, with favorable responses to BTKi; (*ii*) *MYD88*mut*CXCR4*mut, characterized by a longer time to major response, reduced progression-free survival (PFS), and lower response rates to BTKi; (*iii*) *MYD88*wt*CXCR4*wt, more aggressive and associated with poor overall survival (OS) and minimal response to BTKi; and (*iv*) *MYD88*wt*CXCR4*mut, that is rare and likely shares the prognosis of *MYD88*wt*CXCR4*wt [10,40].

Despite the indolent nature of LPL and a median survival exceeding 10 years from diagnosis, 2–10% of patients eventually develop DLBCL, typically exhibiting the non-GCB phenotype, associated with a poor prognosis [4,21,41]. A study on a cohort of 1466 LPL cases found that 20 patients developed DLBCL, with a median OS from transformation of 2.7 years, similar to that of non-GCB DLBCL [4,21]. Similar to CLL evolving into RT, the majority of DLBCL arising from LPL share the same IGHV rearrangement as the dominant LPL clone, but about 25% are clonally unrelated, with 40% of these still deriving from LPL subclones [41]. Due to the dismal outcomes associated with transformed LPL and the limited understanding of the molecular mechanisms of transformation, exploring these mechanisms is essential for developing more effective treatment strategies.

## 2. Molecular Mechanisms in CLL Transformation to RT

Numerous molecular alterations associated with the occurrence of DLBCL-type RT have been identified, while the development of HL-type RT is thought to resemble *de novo* HL and may be linked, at least in part, to Epstein–Barr virus-mediated immunosuppression (Table 1) [42,43,44]. The primary drivers of DLBCL-type RT include genetic lesions, dysregulation of immune checkpoints, and hyperactivation of the B cell receptor (BCR) pathway [45].

### 2.1. TP53 Disruption

**Figure 1:**
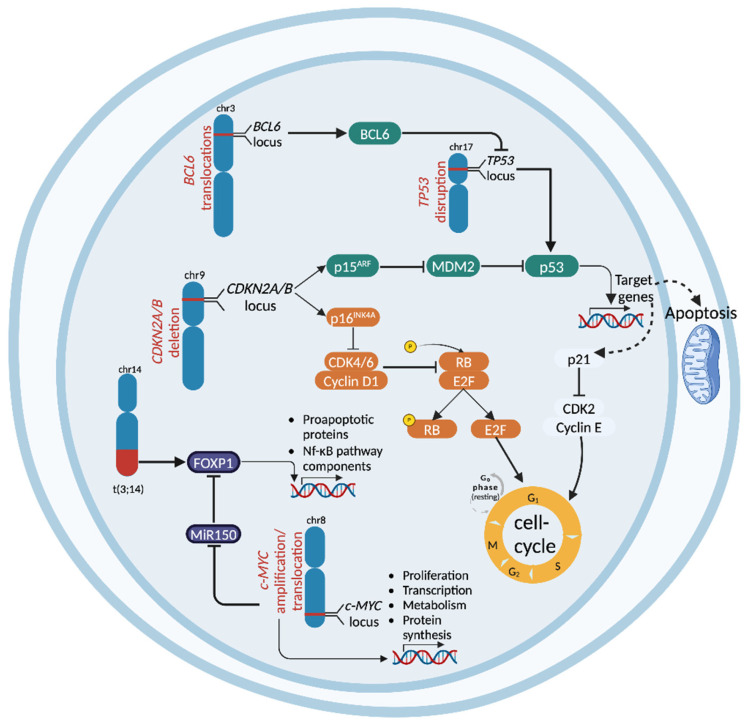
Main chromosomal aberrations implicated in the pathogenesis of HT. Several chromosomal aberrations drive the transformation from indolent to aggressive B cell malignancies. Deletions of the short arm of chromosome 17 disrupt *TP53*, a critical tumor suppressor responsible for DNA damage detection and cell-cycle regulation, contributing to HT into DLBCL-type RT and FL. Similarly, deletions of the short arm of chromosome 9 result in the loss of *CDKN2A/B*, which suppresses the transcription of p16^INK4A^ and p14^ARF^. This disruptive event enhances the G1-S phase transition and promotes MDM2-mediated inhibition of p53, leading to increased proliferation, survival, and reduced DNA damage response, which are key factors in HT across DLBCL-type RT, FL, and SMZL. Translocations involving *BCL6*, frequently observed in tFL, lead to protein overexpression that represses genes such as *TP53*, the DNA damage sensor *ATR*, and the cell-cycle-arrest regulator *CDKN1A*. The t(3;14) translocation involving the *FOXP1* and IGH genes causes FOXP1 overexpression, enhancing proliferation and survival and representing a main driver of chromosomal aberration in the HT of MALT lymphomas. Finally, translocations involving the *c-MYC* locus lead to the hyperactivation of the *c-MYC* transcriptional program, resulting in the overexpression of the c-MYC protein. This overexpression enhances proliferation and survival by promoting the expression of several target genes, including *FOXP1*, leading to DLBCL-type RT and tFL development. Created with *BioRender.com*.

Disruption of *TP53*, through mutation and/or deletion, can either be already present in the CLL phase prior to treatment requirement (in 10–15% of the cases), representing an independent risk factor of DLBCL-type RT development, or can also be acquired at the time of transformation (Figure 1) [46,47]. *TP53* disruption has been observed in a significant proportion of DLBCL-type RT, occurring in 60–80% of clonally related cases and ~20% of clonally unrelated cases [46]. *TP53*, located on the short arm of chromosome 17 (17p13.1), is a tumor suppressor gene that encodes the p53 protein, often referred to as “the guardian of the genome”, playing a crucial proapoptotic role in response to DNA damage [48]. Additionally, p53 contributes to cellular regulation through non-transcriptional mechanisms mediated by protein–protein interactions [49]. By inducing cell-cycle arrest through p21, p53 ensures sufficient time for DNA repair, preventing the replication of damaged DNA [50]. It also regulates essential repair pathways, such as homologous recombination and nucleotide excision repair [51]. Additionally, p53 initiates apoptosis if repair fails, by upregulating pro-apoptotic genes, including *BAX*, *NOXA*, and *PUMA*, while downregulating anti-apoptotic genes, such as *BCL-2*, thereby safeguarding against tumorigenesis [52,53]. Therefore, *TP53* disruption is a pro-tumorigenic event that not only promotes tumorigenesis but also diminishes the cellular response to DNA damage, contributing to chemorefractoriness.

### 2.2. CDKN2A Deletion

Deletion of the cyclin-dependent kinase inhibitor 2A (*CDKN2A*) gene is found in approximately 30% of DLBCL-type RT cases and is often acquired at the time of transformation, suggesting its pivotal role in driving RT in patients harboring this genetic lesion (Figure 1) [54]. *CDKN2A* encodes the cell-cycle regulators p16INK4A and p14ARF, which are responsible for the inhibition of the G1-to-S transition [55]. Under physiologic conditions, cyclin-dependent kinases 4 and 6 (CDK4 and CDK6) drive this step of the cell cycle by activating the E2F-dependent pathway, which, in turn, promotes cell entry into the S phase [56,57]. However, when hyperproliferative signals from constitutively active oncogenes exceed normal thresholds, p16INK4A is activated, inhibiting the activity of CDK4 and CDK6 [58]. Additionally, p14ARF inhibits the MDM2 E3 ubiquitin ligase (a p53 inhibitor), leading to the activation of the p53 transcriptional program, which triggers either apoptosis or cell-cycle arrest [55,59]. Consistently, *CDKN2A* deletion causes the impairment of the negative regulation of cell-cycle progression and resistance to pro-apoptotic stimuli, leading to tumorigenesis. Furthermore, the concomitant loss of *TP53* and *CDKN2A* activity disrupts the balance between positive and negative cell-cycle regulators, both of which are influenced by the BCR signaling pathway [60]. In CLL cells, where BCR signaling is hyperactivated, this imbalance leads to increased proliferation, genetic instability, and risk of HT [1].

### 2.3. NOTCH1 Mutational Activation

**Figure 2:**
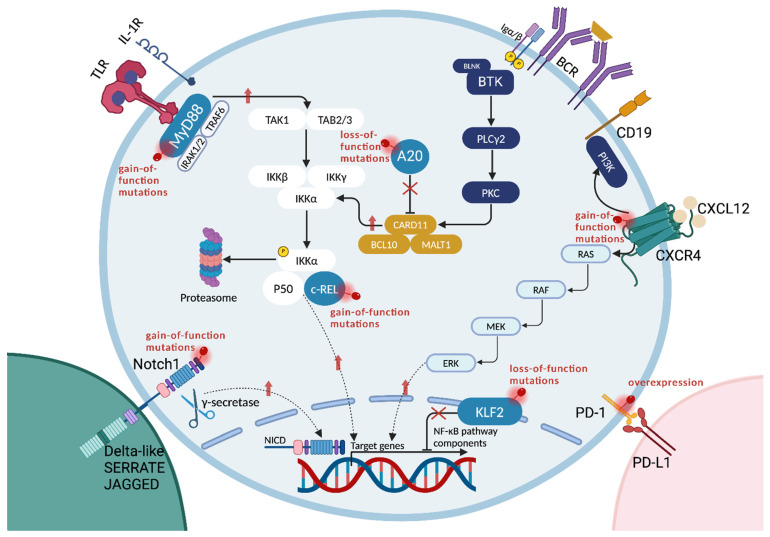
Genetic mutations and microenvironmental alterations implicated in HT. Several molecular pathways implicated in HT are shared among CLL, FL, MZL, and LPL. The NF-κB signaling cascade is frequently involved, driven by gain-of-function mutations in *MYD88* and *c-REL*, as well as loss-of-function mutations in *A20* and *KLF2*. Moreover, the hyperactivation of BCR signaling plays a key role in the pathogenesis of CLL and its progression to DLBCL-type RT. Also, Notch1 signaling is often dysregulated through gain-of-function mutations, leading to the upregulation of genes that promote abnormal cell proliferation and tumorigenesis. Additionally, gain-of-function mutations in *CXCR4* drive the upregulation of the MAPK1 cascade, particularly in LPL translating into a more aggressive biological behavior of the disease. In addition, microenvironmental alterations also contributes to HT. Consistently, the immune checkpoint PD-1 is frequently overexpressed in RT, which further contributes to immune evasion. Created with *BioRender.com*.

*NOTCH1* mutational activation is notably more common at the time of diagnosis of DLBCL-type RT than in CLL, with frequencies of 31% and 8.3%, respectively (Figure 2) [61]. Notch1 is a cell surface receptor activated upon the binding of a ligand of the SERRATE/JAGGED or DELTA families, located on adjacent cells [62]. This binding triggers two sequential proteolytic processes: first, an extracellular cleavage near the membrane by a disintegrin and metalloproteinase, creating a substrate for the second intramembrane cleavage by the γ-secretase complex [62,63]. This results in the intracellular release of the active Notch1 intracellular domain (NICD), which translocates to the nucleus, where it initiates the transcription of various genes that regulate cell proliferation and survival [63]. Under physiologic conditions, this signaling is terminated when specific amino acid sequences (known as degron sites) on the PEST domain are ubiquitinated, leading to the proteasome-mediated degradation of NICD [64]. The most common genetic lesions in DLBCL-type RT are disrupting mutations of the *NOTCH1* PEST domain, impairing ubiquitination and therefore prolonging Notch1 active signaling and promoting abnormal cell proliferation and tumorigenesis [65,66]. Notably, the risk of developing DLBCL-type RT is considerably higher in *NOTCH1*-mutated CLL, with a reported incidence of 45% at 15 years, compared to only 4.6% in *NOTCH1* wild-type CLL over the same period [67]. On these grounds, *NOTCH1* mutations have been identified as a significant risk factor for the development of DLBCL-type RT, with a cumulative risk of developing RT of 45% in *NOTCH1*-mutated CLL [42]. However, these data refer to the CIT era, highlighting the need for updated data in the era of targeted therapies.

### 2.4. c-MYC Abnormalities

The *c-MYC* proto-oncogene and its encoded protein are involved in several essential cellular processes, and abnormalities in this pathway lead to aberrant cell survival, growth, metabolism, self-renewal, and genomic instability (Figure 1) [68]. The *c-MYC* transcriptional program is one of the main targets of Notch1 signaling, as it is directly activated by NCID [69]. In DLBCL-type RT, the primary genetic alterations driving *c-MYC* dysregulation include chromosomal translocations between the *c-MYC* gene, located on chromosome 8, and the IGHV regulatory regions, as well as gene amplification and mutations in the *c-MYC* promoter that enhance its activity. Another mechanism of *c-MYC* dysregulation involves loss-of-function mutations in the *MGA* gene, which normally prevents the c-MYC protein from forming a functional complex with its partner MAX. Together, genetic lesions affecting *c-MYC* and *MGA* contribute to c-MYC dysregulation in approximately 40% of DLBCL-type RT cases [54,70].

### 2.5. BCR Pathway Dysregulation

The hyperactivation of BCR signaling plays a key role in the pathogenesis of CLL and its progression to DLBCL-type RT, as shown in preclinical studies (Figure 2) [60,71,72,73,74]. Two potential models, which likely co-occur, have been proposed to explain this hyperactivation: antigen-dependent and antigen-independent BCR engagement [72,75]. Antigen-dependent signaling occurs when self or foreign antigens bind to the BCR, while antigen-independent signaling arises from Ig-Ig interactions within the same cell membrane, rather than from activating mutations, which are rare [76,77]. Moreover, about 30% of CLL patients share nearly identical VDJ rearrangements of the BCR Ig, forming stereotyped subsets, with subset 8 (IGHV4-39/IGHD6-13/IGHJ5) frequently linked to aggressive disease, trisomy 12, and *NOTCH1* mutations [78,79]. This subset is notably associated with the progression of CLL into DLBCL-type RT, likely due to its heightened reactivity, leading to a significant activation of CLL cells and the selection of more aggressive clones [1,80,81,82]. Moreover, a recent analysis of light-chain variable gene mutational status in CLL identified a significant association between the IGKV1-39/1D-39 rearrangement and *NOTCH1* mutations, trisomy 12, unmutated IGHV, and stereotyped subsets 1 and 8 [83]. These findings suggest a potential role for IGKV1-39/1D-39 in the development of DLBCL-type RT.

### 2.6. Dysregulation of Immune Checkpoints

PD-1 is a surface receptor on T cells that promotes apoptosis of effector T cells and supports Treg survival by interacting with its ligand, PD-L1, which is predominantly expressed on antigen-presenting cells (APCs) such as macrophages, B cells, and dendritic cells (DCs) [84]. As such, the interaction between PD1 and PD-L1 represents one major immune checkpoint inhibitor that regulates tumor escape from immune surveillance (Figure 2) [84]. DLBCL-type RT cells overexpress PD-1, while PD-L1 is primarily found on histiocytes and DCs, thus challenging the traditional PD-1/PD-L1 model of T cell suppression [85,86]. This phenomenon may be linked to the recent finding of PD-1+ regulatory B cells (Bregs) observed in various cancers [87,88,89]. Bregs may suppress the immune system via IL-10 production, causing T cell exhaustion, FOXP3+ Treg expansion, and recruitment of myeloid-derived suppressor cells, or by directly inhibiting T cells through a PD-L1-dependent mechanism, as in thyroid cancer [45,89]. However, the specific role of DLBCL-type RT cells as PD-1+ Bregs requires further study. Additionally, the DLBCL-type RT microenvironment exhibits elevated levels of immune checkpoints LAG3 and TIGIT [45]. LAG3, expressed on activated CD4+ and CD8+ T cells, binds MHC class II on APCs and tumor cells, including DLBCL-type RT cells [90,91,92,93,94]. This interaction transmits inhibitory signals, impairing T cell function and enabling tumor immune evasion [45]. TIGIT, found on natural killer (NK) and T cells, interacts with CD155 on APCs and tumor cells to suppress immune responses [95]. In DLBCL-type RT, CD226, a TIGIT competitor that activates T and NK cells via CD155, is abnormally elevated, suggesting an imbalance favoring activating signals in DLBCL-type RT tumor cells, contrasting with higher TIGIT levels in CLL cells [96,97,98].

The potential sensitivity of DLBCL-type RT to immune checkpoint inhibitors has prompted studies investigating PD-1/PD-L1 axis inhibition. While single-agent PD-1/PD-L1 inhibitors showed limited efficacy, promising results emerged from the phase 2 MOLTO study [45,99]. This trial evaluated a combination therapy of the PD-L1 inhibitor atezolizumab, the BCL2i venetoclax, and the anti-CD20 mAb obinutuzumab, achieving an overall response rate (ORR) of 67.9% [99]. Moreover, targeting BTK could represent a viable treatment strategy due to the overactivity of the BTK pathway [1]. Based on this rationale, the phase II RT1 trial evaluated the combination of the BTK inhibitor zanubrutinib and the PD-1 inhibitor tislelizumab in DLBCL-type RT, yielding promising results with an ORR of 58.3% and a complete response (CR) rate of 18.8% [100]. These findings suggest a potential role for PD-1 and/or BTK inhibitor-based combination therapies in the management of DLBCL-type RT, emphasizing the need for further research to optimize these approaches for improved clinical outcomes.

### 2.7. Novel Molecular Insights in DLBCL-Type RT

Recent studies have revealed additional genetic alterations, including somatic single nucleotide variants (SNVs) and somatic copy number alterations (CNAs), that disrupt pathways involved in DNA repair, chromatin remodeling, MAPK13 signaling, and NF-κB activity, driving DLBCL-type RT development and progression [101]. Specific DLBCL-type RT-related changes include frequent mutations in *IRF2BP2* and SNVs in genes like *CCND3*, and *SRSF1*, alongside several recurrent focal somatic CNAs [102]. Notable findings also highlight the loss of key epigenetic regulators, such as *EZH2*, *DNMT3A*, *KMT2C*, *SETD2*, *TET2*, and *ARID1A*, as well as the c-MYC inhibitor *MGA*, through either mutations or somatic CNAs [17,102]. These alterations underscore critical pathways contributing to DLBCL-type RT, including chromatin regulation, splicing, c-MYC activation, NF- κB signaling, immune evasion, inflammation, apoptosis resistance, DNA damage response, and cell-cycle dysregulation. Together, these events reflect the genetic complexity underlying DLBCL-type RT and emphasize the importance of understanding these pathways to develop targeted therapeutic strategies.

## 3. Molecular Mechanisms in the Transformation of FL

There is no single genetic alteration that solely drives the HT of FL [103,104]. Instead, the transformation process involves a combination of pathogenetic mechanisms affecting cell-cycle control, DNA damage response, proliferation, and microenvironmental changes that are mediated by genetic and epigenetic alterations (Table 1).

### 3.1. CDKN2A/B Deletion

The most frequent genomic aberration acquired during the progression to tFL is the loss of *CDKN2A/B*, occurring in approximately 45% of cases (Figure 1) [30,31,103,105,106]. The loss of *CDKN2A* can also occur in FL, although at a significantly lower frequency (10%), and is associated with poorer clinical outcomes [105]. The loss of *CDKN2A/B* disrupts cell-cycle control, driving unchecked cell proliferation and genomic instability and enhancing cellular aggressiveness [31]. Interestingly, genetic abnormalities in *TP53*, observed in around 20% of cases during tFL, are mutually exclusive with *CDKN2A/B* lesions [31].

### 3.2. c-MYC Abnormalities

Genetic lesions activating the *c-MYC* proto-oncogene, such as chromosomal translocations, gains and/or amplifications, and point mutations, are commonly acquired during the progression to tFL, occurring in approximately 40% of cases (Figure 1) [30,31,107]. Dysregulated c-MYC oncogenic activity can confer multiple advantages to the transformed clone through its pleiotropic roles in promoting cell growth, altering metabolism, and driving genetic instability [31]. *c-MYC* activation can also be driven by epigenetic mechanisms. Consistently, Musilova and colleagues analyzed microRNA (miRs) profiles from serial tFL biopsies and identified five miRs enriched during transformation, including miR-150 [108]. Their findings suggest the existence of a c-MYC/miR-150/FOXP1 axis, where *c-MYC* overexpression represses miR-150 levels, leading to the upregulation of the *FOXP1* gene. FOXP1, a transcription factor crucial for B cell development, is associated with the ABC subtype of DLBCL and is linked to poor clinical outcomes in both DLBCL and FL [108].

### 3.3. TP53 Disruption

*TP53* disruption is uncommon in FL, occurring in approximately 5% of cases [30,109]. In contrast, *TP53* lesions are significantly more frequent in tFL, with an incidence of 25–30% (Figure 1) [107]. An early report by Lo Coco et al. demonstrated that *TP53* mutations were predominantly found in tFL samples compared to baseline FL cases, a finding subsequently validated in larger cohorts [31,107,110]. Studies have shown that *TP53* mutations typically occur without deletion of the other allele in untransformed FL, whereas biallelic alterations through deletion, loss of heterozygosity (LOH), or additional mutations are frequently observed in tFL [31,104]. However, a few studies reported inconsistent results. In a study analyzing 185 diagnostic samples of FL from the Lymphoma/Leukemia Molecular Profiling Project, *TP53* mutations were associated with shorter PFS and OS but unexpectedly showed no correlation with subsequent transformation [109]. Moreover, in that regard, Davies et al. found no direct correlation between *TP53* status and tFL, whereas their study revealed significantly higher MDM2 expression in tFL samples (72%) compared to FL samples (58%) [111]. This suggests that an alternative mechanism disrupting p53 function might be operative in tFL, potentially increasing the transformation risk [111].

### 3.4. BCL-6 Genetic Lesions

B cell lymphoma 6 (*BCL-6*) is a proto-oncogene encoding the BCL6 protein, a crucial regulator of GC development [112]. A key biological function of BCL6 in centroblasts is to promote rapid proliferation while enabling tolerance to genomic damage that occurs during clonal expansion and somatic hypermutation. BCL6 achieves this by directly repressing genes involved in DNA damage detection and checkpoint regulation, including the DNA damage sensor *ATR*, the tumor suppressor *TP53*, and the cell-cycle-arrest gene *CDKN1A* [113,114,115]. *BCL6* is commonly affected by mutations and/or translocations in B cell lymphomas (Figure 1) [116]. Specifically, translocations involving *BCL6* and leading to the overexpression of the protein are characteristic of “double-hit” or “triple-hit” DLBCL, which also include *BCL2* and *c-MYC* translocations [117]. These genetic events are associated with a significantly worse prognosis [118,119]. *BCL6* translocations at the time of FL diagnosis are linked to an increased risk of transformation, with a slightly higher frequency observed at HT (25% in tFL versus 10% at diagnosis) [119]. *BCL6* translocations have been detected in both GCB and ABC tFL [118], whereas in *de novo* DLBCL, they are more prevalent in ABC subtype cases [120].

### 3.5. NF-κB Pathway Dysregulation

Genetic lesions play a significant role in the pathogenesis of tFL by driving the aberrant activation of the NF-κB pathway, a critical signaling cascade that regulates DNA transcription, cytokine production, and cell survival of B lymphocytes (Figure 2) [121]. NF-κB lesions occur in one-third of tFL patients [30]. These alterations include the following: (*i*) The amplification of the proto-oncogene *c-REL* found in 11% of tFL, which encodes the c-Rel protein. c-Rel forms a dimer with p50, a component of the NF-κB complex. c-Rel-p50 dimer translocates in the nucleus and promotes the transcription of other critical components of the NF-κB pathway [30,121]. (*ii*) Loss-of-function mutations or the loss of TNF alpha-induced protein 3 (*TNFAIP3*) are identified in 15% of cases, specifically at HT. TNFAIP3 is a central regulator of inflammatory responses. It negatively modulates the TNF-induced NF-κB proinflammatory signaling pathway, supports B cell survival, and regulates TNF-mediated apoptosis [30,121,122]. (*iii*) Non-L265P *MYD88* mutations are present in both FL and tFL [30,121]. Importantly, 80% of these mutations are restricted to HT samples [30]. *MYD88* encodes the general adaptor protein MYD88, which, following ligand binding, associates to the intracellular domain of interleukin 1 and Toll-like receptors (IL1R and TLR) [123,124]. Then, it recruits the serine-threonine kinase IRAK 4, which, through the activation of IRAK1 and IRAK2 by phosphorylation, leads to the activation of TNF receptor-associated factor 6 (TRAF6) [125]. TRAF6 downstream signaling enhances cell survival by activating the BTK, canonical NF-κB, and MAPK pathways [126].

### 3.6. tFL Cell of Origin

When transformed cases are stratified according to their COO using the Hans algorithm, distinct mutational profiles are observed within each group [21,107,118]. Mutations in *MYD88*, *CD79B*, and *BCL10* are more frequently detected (~15–25%) in ABC-transformed cases, whereas 2p16 (*REL*) amplification is predominantly observed in GCB-transformed cases, consistent with patterns reported in DLBCL [107,121,127,128]. These findings suggest the presence of distinct subgroups of tFL, potentially arising through different transformation pathways, and associated with divergent clinical outcomes. In support of this, a prior study demonstrated increased proliferation rates at HT in a subset of tFL, as indicated by gene expression analysis [129]. This subgroup of tFL was enriched in aberrations of *TP53*, *CDKN2A/B*, and *REL*, highlighting potential mechanistic differences in transformation compared to other tFL cases.

### 3.7. Microenvironmental Alterations

Specific genetic mutations in FL alter the interactions between B cells and their microenvironment. In this context, the herpesvirus entry mediator (HVEM) protein, encoded by the *TNFRSF14* gene, plays a key role in regulating T-cell responses by providing either costimulatory or coinhibitory signals, depending on its interacting ligand [130,131]. The B and T-lymphocyte attenuator (BTLA) ligand, expressed on B cells, interacts with HVEM to suppress T-cell responses [131]. In FL, the *TNFRSF14* gene is frequently affected by mutations (~30–40%), deletions (~20–30%), and/or copy-neutral LOH (~10%) [30,121,132,133,134]. These genetic alterations reduce HVEM expression and enhance BTLA signaling [135,136]. Consequently, *TNFRSF14* aberrations disrupt the HVEM-BTLA inhibitory axis, reshaping the microenvironment of FL and promoting B cell proliferation, activation of lymphoid stroma, and an increase in follicular T-helper cells [136]. Interestingly, a recent study reported that tFL was associated with increased HVEM expression and decreased BTLA expression; however, it did not analyze the mutational status of the two genes [137]. Importantly, the transformation of FL can be further facilitated by acquired immune evasion mechanisms. In particular, these include loss-of-function mutations or deletions in HLA class I components (particularly *B2M*) and *CD58*, which encodes a protein involved in T and NK cell-mediated responses [31,121]. Both proteins are crucial for the recognition of neoplastic cells by the immune system, and their loss can be acquired upon transformation allowing the lymphoma to evade immune detection.

## 4. Molecular Mechanisms in the Transformation of MZLs

MZLs are rare and heterogeneous B cell malignancies, and limited data are available regarding the molecular pathways driving their transformation (Table 1) [138]. This scarcity of information poses challenges to understanding the mechanisms underlying their development and progression. In addition, conflicting data regarding the involvement of specific pathways further complicate this understanding. One such pathway in SMZL is the Notch2 signaling cascade which is affected by activating mutations in 30% of cases [139,140]. The clinical significance of these mutations remains debated. A few studies suggest that *NOTCH2* mutations correlate with better OS and PFS, while others link them to higher relapse, transformation, or mortality risks [139,141]. *NOTCH2* encodes a co-transcription factor that favors B cell differentiation into MZ B cells. Mutations typically truncate the PEST domain, preventing proteasomal degradation and leading to protein accumulation [140,142,143,144].

### 4.1. CDKN2A/B Deletion in SMZL

A recent report shows that, like other lymphomas, the deletion of 9p21 is significantly enriched in transformed SMZL (tSMZL) (Figure 1) [145]. This region contains the tumor suppressor genes *CDKN2A* and *CDKN2B*, which are involved in cell-cycle regulation. Notably, this deletion was the most frequently observed alteration in tSMZL, found in 40.6% of cases, and was absent in SMZL at diagnosis, corroborating findings from previously published studies [140,146,147,148,149].

### 4.2. NF-κB Pathway Dysregulation in SMZL

Regulators of the NF-κB pathway are frequently altered in tSMZL, namely *TNFAIP3* and *KLF2*, mutated in 59.4% and 31.3% of cases, respectively (Figure 2) [145]. Although these genes are frequently disrupted in SMZL, they exhibit a higher mutation frequency in tSMZL [145]. *TNFAIP3* encodes a protein that negatively regulates the NF-κB signaling pathway, through the ubiquitination of NF-κB, leading to its proteasomal degradation [150]. Previous studies have reported that *TNFAIP3* mutations occur in 7% to 15% of cases of SMZL [139,140,151,152]. Notably, Grau et al. showed that truncating loss-of-function mutations in TNFAIP3 are identified in 32% of tSMZL [145]. *KLF2* is a tumor suppressor gene that encodes a transcription factor responsible for repressing NF-κB-mediated B cell activation and their differentiation into marginal zone (MZ) B cells [153]. Loss-of-function mutations and/or the loss of *KLF2* disrupt the protein structure, preventing it from binding to DNA and carrying out its regulatory role [139,147,154,155].

### 4.3. Copy Number Alterations in SMZL

A recent study identified that tSMZL was characterized by a distinct set of driver CNAs, including gains in 1q, 3q, and 18q (encompassing *BCL2*), as well as losses in 1p36 (*ARID1A*), 3p21 (*SETD2*), 7q31-q32 (*KLF2*, *NOTCH2*), 9p21.3 (*CDKN2A/B*), and 13q14.13-q14.3 (*RB1* and *DLEU1/2*) [145]. This profile of genetic alterations largely mirrors that of *de novo* DLBCL, with the exception of 7q loss, a highly specific alteration to SMZL that is rarely observed in other small B cell neoplasms. Furthermore, the study demonstrates that, from a molecular perspective, the genome of tSMZL is significantly more complex than that of SMZL, exhibiting twice the number of alterations [145].

### 4.4. Epigenetic Alterations

The *KMT2D* gene has been found to be mutated in 46.9% of tSMZL [145]. KMT2D is a key epigenetic regulator involved in histone H3 lysine 4 (H3K4) methylation, which is crucial for the transcriptional activation of downstream genes by promoting an open chromatin state [156]. Similarly to *TNFAIP3* and *KLF2*, the frequency of *KMT2D* alterations in tSMZL cases is higher than that reported for SMZL [145]. Additionally, genome-wide epigenetic profiling of SMZL has revealed that aberrant promoter DNA methylation serves as a mechanism of dysregulation affecting critical pathways in the disease contributing to HT [157,158]. Elevated levels of promoter DNA methylation are found in 25% of SMZL cases and are associated with poorer OS and higher risk for HT [157]. Specifically, aberrant methylation appears to contribute to the elevated expression of (*i*) pro-survival genes, such as *TCL1B*, *BCL2A1*, and *FGF1*, as well as members of the BCR and NF-κB signaling pathways (including *CD79B*, *CARD11*, and *PIK3CB*); (*ii*) genes associated with the epigenetic regulator PRC2 complex (*EZH2*, *EED*, and *SUZ12*); (*iii*) genes involved in the JAK-STAT and PI3K/AKT signaling pathways, both involved in cell proliferation [157]. Therefore, aberrant methylation may also play a role in the pathogenesis and/or transformation of SMZL by the inactivation of tumor suppressors and the expression of genes sustaining tumor cell survival and proliferation.

### 4.5. FOXP1 Aberrations in MALT Lymphomas

A multivariate analysis in transformed MALT (tMALT) lymphomas has revealed a significant association between HT and FOXP*1* expression, with FOXP1 detected in five out of eight patients with HT, four of whom exhibited the t(3;14) translocation [159]. The t(3;14) translocation has been implicated in aggressive forms of HT within MALT lymphomas (Figure 1) [159,160,161]. Chromosome 3 harbors the *FOXP1* gene and has been identified as a translocation partner with the *IGH* gene in both DLBCL and MALT lymphomas. Moreover, FOXP1 expression has been recognized as a poor prognostic indicator in DLBCL, particularly associated with the non-GCB subtype [162]. Sagaert et al. examined 70 distinct MALT lymphomas, correlating FOXP1 protein expression with outcome in tMALT lymphomas [159]. Immunohistochemistry and fluorescent in situ hybridization analyses detected FOXP1 expression in approximately 30% of MALT MZL cases, all of which were linked to poor clinical outcomes.

### 4.6. Mechanisms of HT in NMZL

The mechanisms driving the transformation of NMZL are poorly understood due to the rarity of the disease. In a study by Qian and colleagues, six cases of transformed NMZL revealed a significantly higher frequency of del(20q12) compared to non-transformed cases [163]. These transformed lymphomas also showed enrichment in extracellular matrix proteins (COL1A1 and FN1), the growth factor receptor PDGFRβ, the DNA repair protein RAD51, and the signaling molecule WNT11.

## 5. Molecular Mechanisms in the Transformation of LPL

The molecular alterations driving the aggressive transformation of LPL are poorly understood, with *CXCR4* mutations being the primary factors involved (Table 1) [41]. Conversely, *MYD88* L625P gain-of-function mutation, found in the vast majority of LPL, was identified only in 73% of DLBCL transformed from LPL in a recent study [29,38,41]. This finding aligns with previous studies that have identified the absence of *MYD88* mutations as a significant risk factor for the development of HT [164].

Other genetic lesions found at diagnosis or acquired at the time of transformation include mutations in *TP53*, *KMT2D*, *ARID1A*, *BTG1*, *BTG2*, *PIM1*, *CARD11*, and *CD79B*, but the full extent of their role in the transformation remains unclear [41,165,166].

### CXCR4 Mutations

*CXCR4* mutations are found in about 30% of LPL patients overall and in approximately 55% of transformed cases before HT (Figure 2) [41,165]. CXCR4 is a G protein coupled receptor (GPCR) activated upon CXCL12 binding under physiologic condition, functioning as a key factor in directed migration, leukocyte trafficking, and the homing of stem and progenitor cells [167]. In LPL, the most common CXCR4 alteration, occurring in 50% of cases, involves serine 338 (at position 1013), where C > G changes occur in 54% and C > A changes in 25% of cases, both producing a stop codon, while S338 frameshift mutations account for 21% of cases [165]. These mutations create premature stop codons or frameshifts, causing the production of a truncated protein which lacks its regulatory domain, thereby resulting in reduced CXCR4 desensitization and internalization, which leads to prolonged activation following CXCL12 binding [40,165]. The upregulation of the CXCR4 pathway results in abnormally high signaling mediated by diacylglycerol (DAG) and inositol trisphosphate (IP3), which lead to the aberrant activation of several intracellular pathways involved in cell proliferation, growth, and survival, including AKT signaling and therefore the MAPK 1/2 pathway [168]. Consistently, mutations of *CXCR4* translate into a more aggressive clinical behavior in LPL, eventually leading to more symptomatic and progressive disease and ultimately to HT [40].

## 6. Molecular Crossroads: The Shared Pathways Fueling Lymphoma Transformation

Despite the biological and clinical heterogeneity of CLL, FL, MZLs, and WM, the HT of these diseases follows converging evolutionary trajectories driven by common molecular mechanisms (Table 1). These alterations disrupt key tumor suppressor pathways, promote uncontrolled proliferation, and enhance survival signaling, ultimately leading to aggressive disease with increased genomic instability and therapy resistance [1,103,138]. The recurrence of these mechanisms across different B cell malignancies suggests a hierarchical model of transformation, where early genomic lesions, such as *TP53* disruption and *CDKN2A/B* loss, create a permissive environment for further oncogenic events, including c-*MYC* activation and NF-κB pathway dysregulation [1,103,104]. Beyond their individual roles, these alterations reflect an interplay between intrinsic tumor biology and extrinsic selective pressures. *TP53* disruption and *CDKN2A/B* loss not only drive unchecked cell-cycle progression, but also promote resistance to apoptosis, enabling the survival of genetically unstable clones that are prone to further transformation [60,103]. The acquisition of c-*MYC* abnormalities fuels metabolic reprogramming, rapid proliferation, and increased replicative stress, reinforcing tumor aggressiveness [68]. In parallel, NF-κB dysregulation sustains B cell survival through chronic inflammatory signaling and immune evasion, further shaping the tumor microenvironment to support malignant progression [121]. The convergence of these pathways across multiple lymphoma subtypes highlights key vulnerabilities that could serve as both predictive biomarkers and therapeutic targets. Identifying these genetic lesions in the pre-transformation phase could allow for early risk stratification and preemptive therapeutic intervention. Moreover, targeting these pathways through strategies aimed at restoring *TP53* function, inhibiting *c-MYC*-driven oncogenesis, or disrupting NF-κB-mediated survival signaling may hold promise in delaying, or more optimistically preventing, transformation [169,170,171]. Given that many of these pathways also drive resistance to conventional chemotherapy, a precision medicine approach integrating molecular profiling and targeted therapies might improve outcomes for patients at high risk of transformation, shifting the paradigm from reactive treatment to proactive disease management.

**Table 1:** Frequency and biological effect of genetic lesions in HT.

Disease	Gene Lesion	Biological Effect	Frequency Before HT	Frequency at HT	Reference
**CLL**	*TP53*	DNA damage response, cell-cycle regulation	10–15%	60–80%	[46,47]
	*CDKN2A*	Cell-cycle regulation	7%	30%	[54,172]
	*NOTCH1*	NF-κB signaling	8%	31%	[61]
	*c-MYC*	Proliferation and survival	-	30–35%	[54]
	*MGA*	c-MYC inhibition	3%	6%	[70]
	*SETD2*	Epigenetic regulation of gene expression	3%	30%	[173,174]
**FL**	*CDKN2A*	Cell-cycle regulation	5%	45%	[30,31,103,105,106]
	*c-MYC*	Proliferation and survival	5–10%	40%	[30,31,107]
	*TP53*	DNA damage response, cell-cycle regulation	5%	25%	[30,31,107,109,110]
	*BCL6*	B cell differentiation	10%	25%	[119]
	*c-REL*	NF-κB signaling	-	11%	[30]
	*TNFAIP3*	NF-κB signaling	5–10%	15%	[30,121,122]
	*MYD88*	NF-κB/BCR signaling	5%	11%	[31,121]
	*CD58*	Microenviroment dysregulation	-	5	[31,121]
	*B2M*	NF-κB signaling	-	12	[31,121]
	*TNFRSF14*	Microenviroment dysregulation	15–20%	30–40%	[30,121,132,133,134]
**MZLs**	*CDKN2A*	Cell-cycle regulation	5%	40%	[140,146,147,148,149]
	*TNFAIP3*	NF-κB signaling	15%	60%	[145]
	*KLF2*	NF-κB signaling and B cell differentiation	20–25%	35%	[139,145,147,154,155]
	*KMT2D*	Epigenetic regulation of gene expression	10–15%	45%	[157,158]
**LPL**	*CXCR4*	Proliferation and survival	30%	55%	[41,165]

Abbreviations: HT, histologic transformation; CLL, chronic lymphocytic leukemia; FL, follicular lymphoma; MZLs, marginal zone lymphomas; LPL, lymphoplasmacytic lymphoma.

## 7. Conclusions

HT is a major cause of morbidity and mortality in patients with lymphoproliferative neoplasms. Consistently, DLBCL arisen from NHLs still represent an unmet clinical need, due to the high degree of resistance to available treatment approaches. Although no unifying genetic lesion leading to HT has been identified, the central molecular pathways driving transformation include genetic and epigenetic alterations of cell-cycle control, DNA damage response, proliferation, and microenvironmental dysregulation. Importantly, several pathway alterations are shared by indolent B cell malignancies undergoing HT, including *CDKN2A* deletion, *TP53* disruption, *c-MYC* aberrations, and activating genetic lesions of NF-κB signaling. However, the clinical outcome of HT is remarkably different across distinct histologic subtypes, with DLBCL-type RT displaying poorer survival rates, implying that the prognosis relies on the initial lymphoproliferative neoplasm. Although a number of genetic alterations are associated with higher risk of HT, none of the currently investigated biomarkers is able to predict transformation with sufficient precision. In that regard, liquid biopsy, which allows to detect tumor-associated mutations in cell-free DNA in plasma, represents an innovative diagnostic approach to better mirror the spatial and/or intra-tumor heterogeneity [104,169,170]. Therefore, liquid biopsy might serve as a potential tool for transformation prediction. Most of the aforementioned data on the incidence and biology of HT derive from studies conducted in the CIT era, therefore underscoring the need for further investigations in chemo-naïve patients receiving targeted therapy, since they may exhibit distinct biological pathways. Nevertheless, at the present time, a considerable number of HT continue to involve patients with prior exposure to CIT. In light of the fact that the majority of current patients undergoing HT have received CIT, they often develop chemorefractoriness largely due to alterations of DNA damage response mechanisms, which may play a role in HT. On these grounds, targeted therapy directed towards immune checkpoints, BCL2, and BTK, as well as bispecific mAbs and chimeric antigen receptor (CAR)-T cells, have emerged as a promising treatment approach in HT [1,99,100,175,176]. In conclusion, further investigation is necessary for a deeper understanding of the molecular mechanisms underlying HT, in order to further highlight predictive biomarkers of transformation and develop effective treatment strategies.
